# Spontaneous perirenal hemorrhage (Wunderlich syndrome) in the fetus: a case report

**DOI:** 10.1186/s13256-023-03867-4

**Published:** 2023-05-06

**Authors:** Yongmei Jia, Junxia Gao

**Affiliations:** Xiangyang Central Hospital, Hubei University of Arts and Science, Xiangyang, 441021 China

**Keywords:** Spontaneous perirenal hemorrhage, Hydronephroses, Bladder dysfunction, Ultrasound

## Abstract

**Background:**

Spontaneous perirenal hemorrhage (Wunderlich syndrome) in the fetus is a rare urinary system disease. Prenatal ultrasound diagnosis presents challenges due to the lack of specific clinical features.

**Case presentation:**

A 27-year-old Chinese woman gravida 2 para 0 found her fetus with the left Wunderlich syndrome accompanying bilateral hydronephroses and bladder dysfunction with an early diagnosis through prenatal ultrasound and postnatal magnetic resonance imaging. After a timely emergency cesarean section, the infant was administrated antimicrobial prophylaxis and an indwelling catheter treatment. Ultrasound follow-up showed his urinary system gradually developed normally.

**Conclusion:**

A fetus with bilateral hydronephroses accompanying bladder dysfunction should be observed because of the risk of spontaneous renal rupture with hemorrhage formation. Ultrasound and magnetic resonance imaging play a vital role in the diagnosis and follow-up of Wunderlich syndrome. Early diagnosis facilitates better pregnancy planning and appropriate care of newborns.

## Background

Wunderlich syndrome (WS) is a relatively uncommon and potentially life-threatening urinary disease that is typically characterized by acute abdominal pain, palpable mass, and hypotensive shock. A comprehensive analysis of articles from 2000 to 2022 shows that WS has been reported only in adults and not in fetuses [1, 2]. Herein, we report a case of the left WS accompanying bilateral hydronephroses and bladder dysfunction with an early diagnosis through ultrasound and magnetic resonance imaging (MRI). Timely prenatal diagnosis was very important for pregnancy management and postpartum care.

## Case presentation

**Fig. 1:**
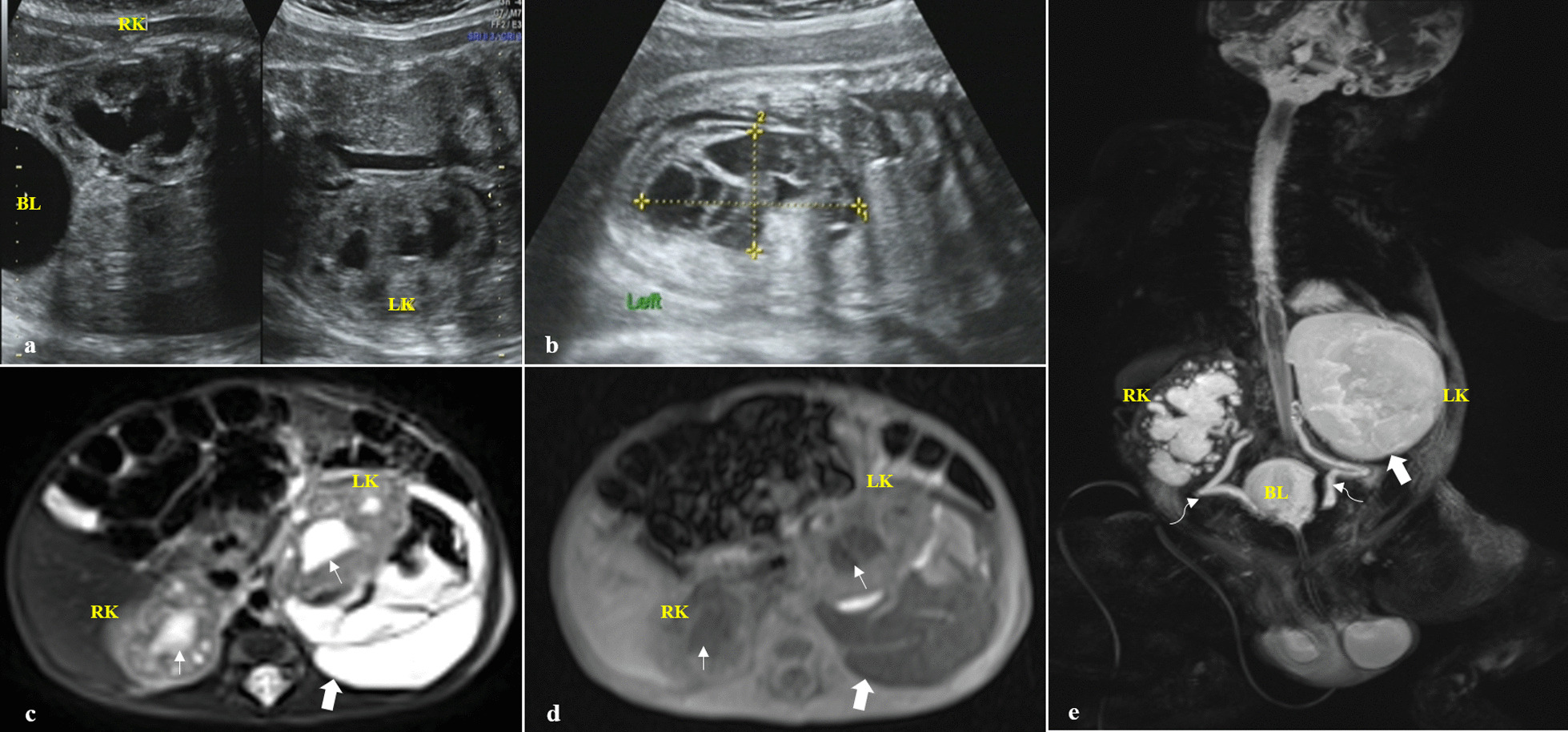
**a** and **b** Coronal view of ultrasound showing bilateral hydronephrosis and the left WS at 36 weeks of gestational age. **c** and **d** Cross-weighted images of T2 and T1 by postnatal infant MRI demonstrating bilateral hydronephrosis and WS, respectively. **e** MRU imaging of the urinary system revealed a thickened urinary bladder and bilateral ureteral dilatation. Thick and thin arrows show WS and hydronephrosis, respectively. Curved arrow showing ureter dilatation

**Fig. 2:**
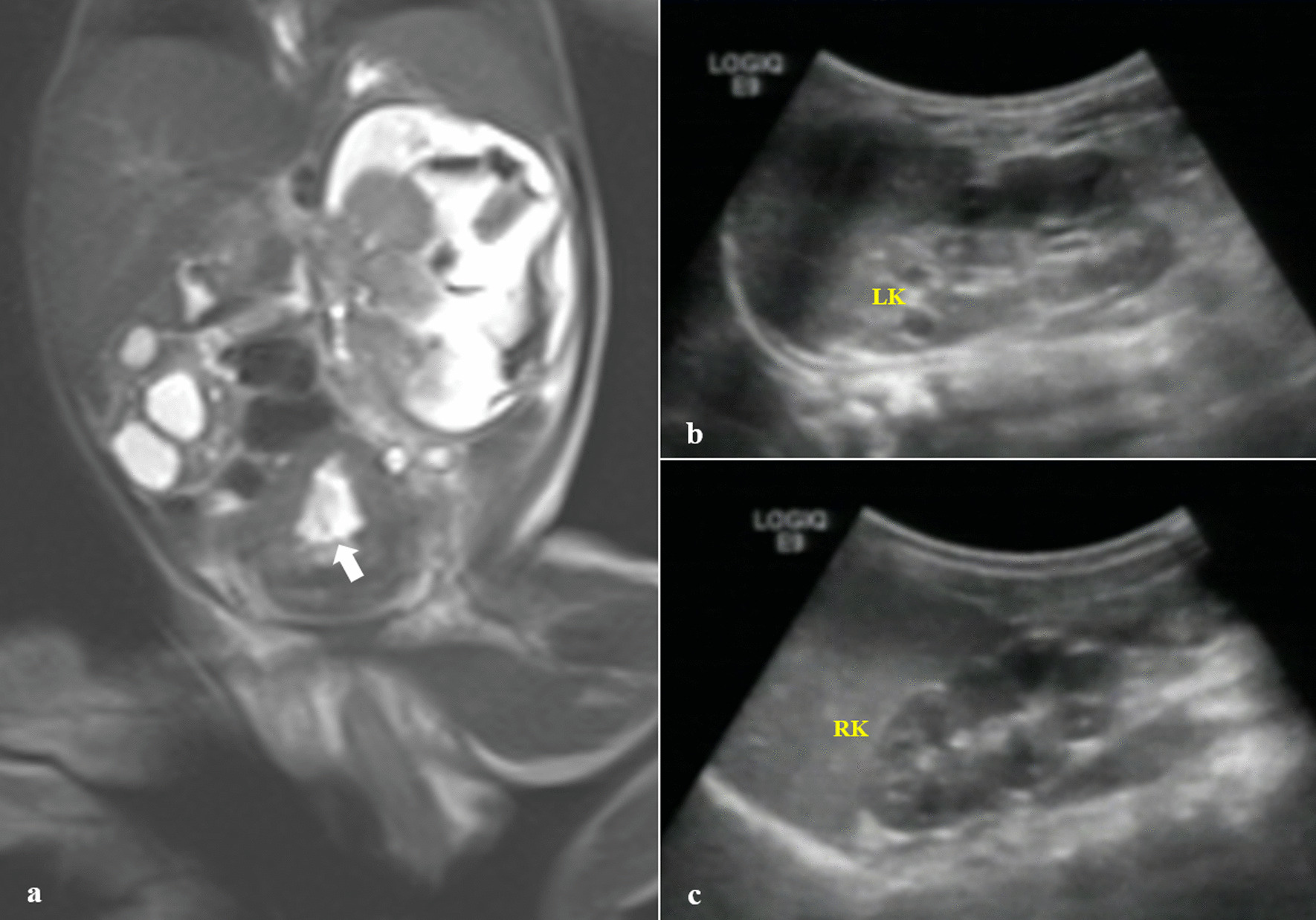
**a** A T1-weighted coronal view revealed a bladder wall irregularity and thickening. **b**, **c** Ultrasound images of both kidneys at 8 months. Thick arrow showing bladder

A healthy 27-year-old Chinese woman gravida 2 para 0 with an established spontaneous pregnancy was referred to our department with the diagnosis of fetal bilateral kidney dysplasia at another center. Fetal morphology scan, nuchal translucency scan, and Down’s syndrome screening had been reported as normal. She had no relevant medical, family, or psychosocial history. At 36 weeks of gestation, the ultrasound revealed enlargement of bilateral kidneys, hydronephrosis, and dilated bladder. The size of the left perirenal effusion was about 64 × 34 mm, which significantly displaced the left kidney anteriorly. Multiple light bands, flocculent echo, and slightly higher echo mass in the effusion suggested WS (Fig. 1a, b). Additionally, oligohydramnios was noticed. An emergency cesarean section delivered a male infant with a weight of 2530 g. Apgar score was 8, 8, and 10 at 1, 5, and 10 minutes, respectively. Vital signs were stable. No anomaly was detected on general physical examination. No abnormality was seen in the blood and urine routine analysis. His serum creatinine of 156.1 μmol/L indicated impaired renal function. Postnatal MRI confirmed the left WS, which was mainly with long T1 and T2 signals mixed with short T1 and short T2 signals and light bands (Fig. 1c, d). Magnetic resonance urography (MRU) revealed a bladder wall irregularity and thickening, bilateral ureteral dilatation, and urinary retention (Figs. 1e, 2a), which were considered bladder dysfunction. The patient was administered antimicrobial prophylaxis and an indwelling balloon catheter due to urinary retention. After 30 days of conservative treatment, he was able to urinate on his own after it was removed. Ultrasound showed that the diameter of WS was about 20 mm at 2 months and disappeared at 8 months (Fig. 2b, c; Table 1). His urinary system was developing normally at 24-month follow-up periods.

**Table 1:** Change in diameter of left WS during 24-month follow-up periods

Time (month)	Diameter of the WS (mm)
0	64
2	2
8	0
24	0

## Discussion

Spontaneous WS in the fetus is a rarely reported urological condition. WS was first proposed by Wunderlich in 1856, which referred to an encapsulated accumulation of renal blood into the perinephric spaces or subcapsular [3]. The differential diagnosis of cystic masses located between the kidney and spine includes urinoma, lymphangioma, mesenteric cyst, and so on. In this case, WS needed to be differentiated from urinoma, which was a poor prognostic factor for renal function [4]. Multiple slightly strong echoes, septations, and flocculent echoes were more likely to be seen in ultrasonography of hematoma. The presence of characteristic high-intensity signals of acute hematoma on T1-images may tend to consider hematoma rather than urinoma.

The factors contributing to WS include renal tumors, angiomyolipoma, renal arteriovenous malformation, hydronephrosis, tuberous sclerosis complex, and so on [1–3, 5]. Literature research indicated that renal tumors were the most common cause of WS, and hydronephrosis was an uncommon cause [1, 3, 6]. In the present case, hydronephrosis was believed to be the direct cause of WS. The infant MRI and MRU revealed bilateral hydronephrosis, hydroureter, and bladder dysfunction. Several clinical studies have shown that incomplete detrusor/sphincter coordination of the bladder may be a normal pattern of immature urination dynamics in newborns, resulting in functional bladder outlet obstruction of various degrees [7–9]. The uncoordinated pattern of micturition can also be found in late fetal development, most of whom are male [10]. Vesicoureteral reflux was associated with hydronephrosis, and various bladder dysfunction such as detrusor instability and bladder/sphincter dyssynergia [7, 9–11]. The abnormal urine volume increase in the left renal collecting system may place pressure on the left renal parenchyma, leading to the thinning of the renal parenchyma, or even the rupturing of small blood vessels in the renal parenchyma and perirenal hematoma formation. With the growth of infants, the coordination function of the bladder detrusor/sphincter matured, hydronephrosis and hydroureter gradually disappeared, and WS was also absorbed at 24-month follow-up periods.

Ultrasound, computerized tomography (CT), and MRI are the main imaging tools to identify the underlying cause and to develop appropriate treatment methods to reduce significant morbidity [12, 13]. Possible treatment for WS includes conservative treatment, vascular embolism, nephron-sparing surgery, or radical resection. In our case, his hemodynamics and hemoglobin were at normal levels, so conservative treatment was implemented. After conservative treatment with antimicrobial prophylaxis and an indwelling balloon catheter, the patient’s renal function gradually returned to normal.

## Conclusion

We describe a relatively rare case of spontaneous WS accompanying bilateral hydronephroses and bladder dysfunction in the fetus. Ultrasound and MRI play a vital role in the diagnosis and follow-up of WS. Early recognition of WS should lead to better pregnancy planning and providing the appropriate care for newborns.

## Data Availability

Data are available upon reasonable request. All data relevant to the study are included in the paper or uploaded as additional material.

## Abbreviations

WS: Wunderlich syndrome
CT: Computerized tomography
MRI: Magnetic resonance imaging
MRU: Magnetic resonance urography
